# Opportunities of Wearable Technology to Increase Physical Activity in Individuals with Chronic Disease: An Editorial

**DOI:** 10.3390/ijerph16173124

**Published:** 2019-08-28

**Authors:** Jennifer L Scheid, Sarah L West

**Affiliations:** 1 Department of Health Promotion, Daemen College, 4380 Main St, Amherst, NY 14226, USA; 2 Department of Biology & Trent/Fleming School of Nursing, Trent University, Peterborough, Ontario, ON K9J0G2, Canada

**Keywords:** chronic disease, wearable technology, self-monitoring, accuracy, physical activity

## Abstract

In this editorial, we will discuss one promising tool to encourage physical activity participation in individuals with chronic disease: The use of wearable technology.

In this Special Issue on Exercise and Chronic Disease in *IJERPH*, contributions that examine the role of physical activity (PA) on health in chronic disease were invited. While there continues to be much-needed research published that characterizes the importance of PA in individuals with chronic disease, there is still the question about how researchers and the healthcare team can work to increase PA participation rates in the context of chronic disease. In this Editorial, we will discuss one promising tool to encourage PA participation: The use of wearable technology.

Wearable technology is extremely popular and was named the #1 fitness trend in 2019 by the American College of Sports Medicine’s *Health and Fitness Journal* [1]. Wearable technology for the quantification of human movement typically includes devices worn on the wrist, chest, arm, hip, or any other part of the body that can measure PA by movement, heart rate, or global positioning system (GPS). One very common and increasingly popular choice of wearable technology is the fitness watch. Fitness watches, by companies such as Apple, Fitbit, Garmin, Misfit, and Polar, include a lot of capabilities. For example, most fitness watches have integrated accelerometers that quantify movement in multiple directions, but upgraded fitness watches also employ heart-rate monitors, altimeters, and global positioning systems (GPS) to better track daily activity and other indices of health. Combined, this information can provide accurate and detailed information about daily PA.

PA is associated with many health benefits, and there is evidence for a dose-response relationship between PA participation and improved cardiorespiratory, metabolic, musculoskeletal, and mental health [2]. PA participation is also associated with a decreased burden of disease, such as colon and breast cancers, diabetes, and osteoporosis among others [3,4]. Beyond disease prevention, increased PA is independently associated with a decrease in all-cause mortality in adults [5]. Thus, the evidence is strong that participation in PA not only improves health and prevents, treats, and/or alleviates many chronic diseases, but it can also be used to increase longevity and quality of life. Despite this, individuals with chronic disease often participate in low levels of PA; often, they do not meet international guidelines for suggested amounts of PA, and compared to healthy controls, chronic disease populations have worse PA engagement [6]. There remains a disconnect between the research demonstrating the benefits of PA in individuals with chronic disease and the uptake of PA behavior.

Wearable technologies are continuously improving, by integrating with smartphones and their associated apps and platforms more seamlessly. They are also becoming more fashionable and, thus, desirable to wear. For example, Fitbit and Apple not only offer features such as integrated GPS, heart-rate monitoring, sleep insights, and food-intake monitoring that sync with a smartphone, these companies also offer fitness watches with interchangeable watch faces and watch bands. Data highlighting the growing popularity of wearable technology are beginning to emerge; for example, a representative sample of 1215 adults from Canada indicated that 38% of people have access to a physical activity-tracking device [7]. In a sample of Finnish adolescents, 27% (1190/4413) of the adolescences owned a smartwatch or a heart-rate monitor [8]. Even older adults report using technology to monitor PA; in one survey of adults (≥50 years of age) in Switzerland, it was found that 20.5% used mobile devices to keep track of PA levels [9]. Thus, wearable technology may be a tool to help increase physical activity and improve health in persons with chronic diseases. In assessing the utility of wearable technology (more specifically, fitness watches) in persons with chronic disease, we will briefly examine the following questions: (1) Can a fitness watch help someone with a chronic disease increase their physical activity? (2) How does a fitness watch help someone with a chronic disease improve their physical activity? (3) What are some important considerations and what research needs to be conducted to help us better understand the relationship between fitness watch use and chronic disease management?

## 1. Can a Fitness Watch Help Someone with a Chronic Disease Increase Their Physical Activity? 

In otherwise healthy populations, wearable technology has been demonstrated to increase daily PA levels, and the ability of wearable technology to increase PA seems to vary based on individual factors including lower baseline PA [10], meaning that a lower baseline PA is associated with an increased benefit of using wearable technology to increase PA. Since the PA levels of chronic disease populations are often reported as lower than that of healthy populations, there may be an opportunity to increase PA using wearable technology to a greater degree than has been demonstrated in healthy populations.

Current research suggests that fitness watches can increase PA and could, theoretically, be used in combination with other medical recommendations to improve health parameters in those with chronic disease. For example, activity monitors (or fitness watches), in combination with other lifestyle interventions, appear to be successful at increasing PA in obese men and women [11], depressed and alcohol-dependent women [12], and breast cancer survivors [13]. In one study, authors examined the ability of a fitness watch to increase moderate to vigorous PA in overweight/obese adults over six weeks, while also examining if the addition of encouraging/reminder text messages (SMS) to a participant’s cell phone would further influence PA. This study found that the use of the fitness watch increased moderate to vigorous PA, but that the addition of the SMS messages did not impact PA participation significantly beyond one week of follow-up [14]. Thus, fitness watches might independently be a potent way to increase PA. Other pilot studies have demonstrated the feasibility of fitness watch use in childhood cancer survivors [15,16] and pediatric juvenile idiopathic arthritis patients [17]. These few studies demonstrate that increases in steps, total daily PA, and moderate/vigorous PA are possibly facilitated by fitness watch use, but that fitness watches may need to be used in combination with behavioral interventions that include some type of encouragement to increase PA and goal setting. 

## 2. How Does a Fitness Watch Help Someone with a Chronic Disease Improve Their PA?

Fitness watches allow users to see their personal, real-time PA by having constant access to this information on their wrists, and, therefore, employ self-monitoring of PA. Self-monitoring is common in many lifestyle interventions that focus on increasing PA or changing nutrition and food intake. Self-monitoring one’s food intake can have a very beneficial effect on healthier eating habits and has been reported to improve success during weight-loss interventions [18]. Theoretically, the individual can then make a purposeful decision to do more PA, e.g., go for a walk on their lunch break, because they have a greater awareness about their daily activity. 

Technology (fitness watches and activity trackers) work to increase the user’s awareness of their actual daily PA, and if they are motivated to change their behavior (such as by direction via their healthcare team), the individual can increase their activity to achieve predetermined PA goals. By using self-monitoring techniques that provide awareness of PA, use of a fitness watch can help someone improve their PA. This motivation to increase PA with wearable technology has been demonstrated in individuals with chronic disease as well; one recent study conducted a qualitative analysis in 29 participants with chronic disease, and found that participants thought the use of wearable technology (in this case, an activity tracker) motivated them to be more active [19]. 

Fitness watches may help someone improve PA based on many different individual factors. To this end, Ellingson et al. [10] demonstrated in healthy individuals that activity trackers can have beneficial effects on PA, but these results varied based on the individual. For example, individuals with low initial PA were able to increase PA on their own when they were given activity trackers. However, individuals with higher initial PA were more successful at maintaining their PA with the addition of motivational interviewing and habit education. The results of this study further highlight that while fitness watches may have some impact on PA, they may need to be used in combination with behavioral interventions customized to the individual.

In addition to the behavioral benefits of having real-time feedback to help with goal setting and self-monitoring, fitness watches and activity trackers might be useful tools to help individuals meet exercise prescriptions set by a medical or exercise professional. Recently, McNeil et al. [13] demonstrated the ability to increase PA in 45 adult cancer survivors using different prescribed exercise intensities, including lower-intensity PA (300 min·week^−1^ at 40–59% of heart-rate reserve) or higher-intensity PA (150 min·week^−1^ at 60–80% of heart-rate reserve). These intensity differences were successfully tracked using Polar 360 activity trackers [13]. Optimal exercise prescription to improve health includes the FITT-VP principal that outlines exercise guidelines in patients considering frequency, intensity, time, type, volume, and progression in prescribed exercise programs [2]. Wearable technology that includes heart-rate tracking can track the intensity of PA in addition to frequency and total time of exercise sessions [13]. Therefore, a fitness watch can improve motivation and accountability, be part of a multi-approach program aimed at increasing PA, and can help provide PA intervention-specific feedback to the participant and prescriber.

## 3. What are Some Important Considerations and What Research Needs to be Conducted to Help Us Better Understand the Relationship between Fitness Watch Use and Chronic Disease Management?

We note that while data seems to support that wearable technology positively impacts PA in chronic diseases, the use of fitness watches and other devices in the context of chronic disease is not without complexities to consider. We have mentioned the necessity of individualized PA prescription in those with chronic disease, and that the use of wearable technology may in fact facilitate this. An individualized approach to managing someone with chronic disease is a necessity, and the same needs to be considered when we prescribe PA using technology. For example, a prescription to increase moderate to vigorous PA by 30 min in everyone with type 2 diabetes is not appropriate. There is a wide range of symptomology within each chronic disease that needs to be considered before we develop PA interventions, with the assistance of wearable technology or not. For example, in those with type 2 diabetes, clinical symptoms can include mild to severe peripheral neuropathy, skin weakness, and open sores, and the consideration of co-morbidities such as chronic kidney disease or hypertension. Therefore, while wearable technology can be a tool used to increase PA in chronic disease, there are many challenges associated with chronic disease PA prescription that are not addressed by the use of technology alone. Population characteristics associated with chronic diseases may also impact uptake of wearable technology use; for example, older individuals may be less open to (or less likely to) incorporate technology to increase PA [9]. Therefore, diseases that affect older individuals more commonly (such as chronic obstructive pulmonary disease) may be more difficult to target with technology due to differences in technology readiness and literacy. It is important to acknowledge that increasing PA, even with the use of wearable technology, will likely remain a large challenge. 

As previously discussed, some published research has used fitness watches or similar activity monitors in combination with other lifestyle interventions to increase PA in those with chronic diseases or conditions [11,12,13,15,16]. However, many of these studies include a small sample size and no control groups. For example, Abrantes et al. piloted an intervention using Fitbit activity monitors to strategically increase PA in a small group of 15 women with depression, but did not include a control group in their research design [12]. Despite the small sample sizes and the lack of control groups in some of these studies [12,15], published pilot research has supported that individuals with chronic disease are receptive to using fitness watches to track their PA and meet PA goals. Current wearable technology offers patients many ways to monitor their PA; step count, the number of calories burned through energy expenditure, the amount of time spent sedentary, or number of minutes of exercise are all popular ways to monitor PA. However, how each of these measures are calculated (i.e., the equations used) and/or the accuracy of each of these measures can vary by device. Therefore, future studies should validate these measurements in a variety of devices, in populations with chronic disease, to determine which measures most accurately reflect PA participation.

In addition to examining the type of variables used to track activity, future research also should include high-quality randomized controlled trials that include standard lifestyle interventions with and without fitness watches to truly determine if the technology is increasing adherence to PA recommendations. A meta-analysis exploring activity watches in obese populations by de Vries et al. [11] suggests that behavioral weight-loss interventions employing activity watches (or fitness watches) increase PA more than usual care [11]. However, this meta-analysis was unable to determine if a behavioral weight loss-intervention using activity watches to help self-monitor could increase PA levels more than behavioral interventions without activity watches [11]. Research needs to focus on examining the use of wearable technology in individual populations with chronic disease, and to examine the impact on PA as an outcome as well as integration of other health outcomes (such as blood pressure, quality of life, and longevity). 

Not only do we need more research to determine if wearable technology can increase PA and improve the health of people with chronic diseases, feasibility analyses to determine if wearable technology (with the goal to increase PA) should be implemented into routine clinical practice are still required. This research should focus on the economic and operational feasibility of integrating wearable technology into clinical practice. There are many logistical considerations that would need to be addressed, for example online portals that need to be available so that members of an individual’s healthcare team can access the information collected (while still protecting the privacy of this information). We also need to consider what features of the wearable technology resonate with people living with chronic disease, and address if more specific technology should be developed for these populations. 

Since the majority of current wearable technologies are not designed to increase PA in patients with chronic conditions, medical providers, such as doctors and nurses, in combination with exercise professionals that work with patients with chronic disease, need to understand how goals can be manipulated using fitness watches. The standard goals of daily PA prescribed to the general healthy population are not always appropriate for chronic disease (i.e., 150 min of moderate-vigorous PA per week, and/or 10,000 steps per day); in fact, evidence suggests that clinically meaningful changes in health may be attained with lower amounts of PA [20]. Nevertheless, these goals are easy to manipulate through the fitness watch applications, making individualized PA prescription possible. Therefore, there is a necessary learning opportunity for both individuals with chronic disease who can use smartwatches to improve PA, as well as the healthcare team that wants to use the fitness watch as a prescription tool. However, there is still a lack of high-quality research supporting the use of wearable technology to improve PA and other health outcomes in various chronic diseases. 

## 4. Conclusions

We know that PA is beneficial at improving a whole range of symptoms associated with chronic diseases (i.e., poor physical function), at chronic disease prevention (i.e., obesity or cardiovascular disease), and even treating certain chronic diseases (i.e., type 2 diabetes). Despite this knowledge, we still struggle to engage individuals with chronic disease in PA participation. While there continues to be research that examines how PA improves health in chronic disease, and what PA prescription results in the best improvement in health with the least adverse effects, there is a gap in the published literature with regards to high-quality intervention delivery. Wearable technology is a potential tool to facilitate PA prescription and might be an appropriate tool to use in the setting of chronic disease. High-quality, disease-specific randomized controlled trials that examine many outcomes are still needed; but, despite the current challenges, we believe that the future of the use of wearable technology in PA prescription is certainly one that is exciting and encouraging.
